# Spherulization as a process for the exudation of chemical cues by the encrusting sponge *C. crambe*

**DOI:** 10.1038/srep29474

**Published:** 2016-07-06

**Authors:** Eva Ternon, Lina Zarate, Sandrine Chenesseau, Julie Croué, Rémi Dumollard, Marcelino T. Suzuki, Olivier P. Thomas

**Affiliations:** 1 Université Nice Sophia Antipolis, Institut de Chimie de Nice, UMR 7272 CNRS, Faculté des Sciences, Parc Valrose, 06108 NICE Cedex 2, France; 2 Sorbonne Universités, UPMC Univ Paris 06 and CNRS, Laboratoire de Biologie du Développement de Villefranche-sur-mer, Observatoire Océanologique, F-06230 Villefranche-sur-Mer, France; 3 Aix Marseille Univ, Univ Avignon, CNRS, IRD, IMBE, Institut Méditerranéen de Biodiversité et d’Ecologie marine et continentale, Station Marine d’Endoume, rue de la Batterie des Lions, 13007 Marseille, France; 4 Sorbonne Universités, UPMC Univ Paris 06 and CNRS, Laboratoire de Biodiversité et Biotechnologies Microbiennes (LBBM), Observatoire Océanologique, F-66650 Banyuls-sur-Mer, France; 5 National University of Ireland Galway, Marine Biodiscovery, School of Chemistry, University Road, Galway, Ireland; 6 Université Côte d’Azur, CNRS, OCA, IRD, Géoazur, 250 rue Albert Einstein, 06560 Valbonne, France

## Abstract

Ecological interactions in the marine environment are now recognized to be partly held by chemical cues produced by marine organisms. In particular, sponges are sessile animals thought to rely on the bioactive substances they synthesize to ensure their development and defense. However, the mechanisms leading the sponges to use their specialized metabolites as chemical cues remain unknown. Here we report the constant release of bioactive polycyclic guanidinic alkaloids by the Mediterranean sponge *Crambe crambe* into the dissolved and the particulate phases using a targeted metabolomics study. These compounds were proven to be stored into already described specialized (spherulous) sponge cells and dispersed into the water column after release through the sponge exhaling channels (oscula), leading to a chemical shield surrounding the sponge. Low concentrations of these compounds were demonstrated to have teratogenic effects on embryos of a common sea squirt (ascidian). This mechanism of action called spherulization may therefore contribute to the ecological success of encrusting sponges that need to extend their substrate cover to expand.

Marine sponges are key components of marine ecosystems and members of these sessile invertebrates can be found in all marine habitats from tropical to polar latitudes and from littoral to abyssal depths. The ecological success of some marine sponges has in part been attributed to the ability of these organisms or their associated microbiota to biosynthesize a large diversity of metabolites^1^,^2^. Indeed, during their long evolutionary history, marine invertebrates and especially sponges have developed rather specific metabolisms leading to low molecular weight and bioactive substances. Due to high structural complexity and originality^3^, these specialized metabolites have mostly been investigated for their use in the pharmaceutical industry. From an ecological perspective, those compounds were evoked to be involved in ecological interactions^4^ such as defense against potential predation^5^,^6^,^7^,^8^, competition for substrate^9^,^10^, deterrence or fouling by other organisms^9^,^11^,^12^ and may even structure marine ecosystems^13^.

Until recently, it was assumed that most of the chemical interactions were operating through contact in the inner part of the sponge (antimicrobial) or the outer part (deterrence). However, due to their large propensity to modify the composition of the seawater linked to a high filtering activity, sponges chemical interactions could also occur through expelling chemical cues to their surroundings. Indeed, chemical cues conveyed in the seawater are now commonly thought to be involved in the interactions between marine organisms^14^,^15^,^16^,^17^. Direct observations of exudation of allelochemicals by sponges are scarce and restricted to some pioneering studies performed by the group of Thompson. They were able to detect bromotyrosine alkaloids in the non-soluble material coming off individuals of the suspended tubular sponge *Aplysina fistularis*^18^,^19^. Surprisingly this result was not followed by a clear characterization of the mode of transport and action of these allelochemicals in the environment.

We decided to address in detail the question of the exudation of chemical cues in the surrounding seawater by marine sponges using *Crambe crambe* (Schmidt, 1862) as a model. The “toxic” sponge *C. crambe* has been extensively studied both in terms of ecology and chemical content. The specialized metabolites biosynthesized by this sponge are mainly constituted by a large diversity of bioactive polycyclic guanidine alkaloids (PGA)^20^,^21^,^22^,^23^. They are distributed in two main chemical families called crambescins with one or two cycles and crambescidins with five heterocyclic rings (Fig. 1). Several crambescidins (named from their molecular weight; 800, 816 and 830) have been isolated and characterized^21^,^22^,^24^ together with three sub-families of crambescins A, B and C which differ by the presence of a pyrrolidine ring (A), a spiroaminal (B) and a linear 3-hydroxypropyl chain (C) respectively^20^,^23^,^25^. In terms of pharmaceutical applications both crambescins and crambescidins exhibited substantial biological activities. Crambescidin 800 induces differentiation of K562 leukemia cells^26^ and displays antifungal and antiviral activity^27^. Crambescidin 816 exerts a Ca^2+^ antagonist activity in neurons^28^,^29^,^30^, reduces human colon carcinoma cells viability^30^, and this compound is cytotoxic against several human tumor cell lines^30^ and cortical neurons^31^. Crambescins also showed some activity on ionic channels^32^. Considering their biological activity and relatively high concentrations in sponge tissues, both crambescins and crambescidins are likely to be involved in the chemical mediation with other marine micro- and macro-organisms^6^,^33^. PGA present in the endometabolome may therefore exhibit key roles as antimicrobial agents against pathogens but also at the surface of the sponge as antifoulants. Focusing on the exometabolome of this sponge we wanted to better understand the presence, mode of action as well as possible ecological significance of PGA released in the immediate surrounding environment. We therefore attempted to address the following questions in this work: 1) can we detect some PGA in the sponge surroundings? 2) if yes, can we identify the mode of transfer of these compounds into the seawater, 3) what are the effects of the different compounds present in the exometabolome on the larval development of competitors? The exudation process was studied using a PGA targeted metabolomic approach by UHPLC-HRMS on the dissolved and particulate phases of the surrounding seawater above the sponge. In addition, recent studies of the microbiome of *C. crambe* have demonstrated the presence of bacteria in the mesohyle of *C. crambe*^34^, as well as the dominance of the microbiome by phylotypes belonging to an uncultivated group in the Betaproteobacteria. This finding challenged the previous notion that this sponge was devoid of endosymbionts, and opened the possibility that some of the PGA might have a bacterial origin^35^. Microscopic observations of the exuded seawater allow us to associate the putative released compounds with specific cells or micro-organisms. Finally, the observation that some specialized metabolites are naturally present in the seawater surrounding the sponge will raise the possibility of allelopathic interactions with other marine organisms and then the presence of true chemical mediation. In a complementary manner of previous studies on the chemical ecology of this sponge and in order to assess the contribution of exuded PGA to the ecological success of this species, we investigated the effects of two PGA on embryonic development of the Mediterranean tunicate *Phalusia mamillata*. Adult ascidians have a swimming planktonic stage and the tadpole larva must settle on the benthos to metamorphose into a sessile organism^36^. Any developmental malformation preventing the formation of a motile larva will impair settlement and metamorphosis thereby reducing the ecological/reproductive success of this species.

## Results

### Specialized metabolites are constantly released from *C. crambe* trough a natural process

**Figure 2:**
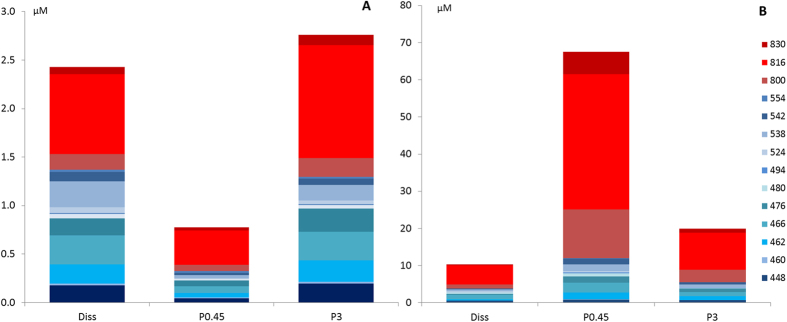
Amount of each targeted metabolite (μM) in the exometabolome, (**A**) for natural samples and (**B**) for stressed samples. (Diss): dissolved form, (Porosity of the filter in μm): particulate forms. The number associated to each color corresponds to the molecular mass (M) of each targeted specialized metabolites (see Table 2).

The exometabolome was analyzed by collecting the seawater *in situ* at 1 cm above healthy specimens of around 50 cm^2^ *C. crambe* in a 1L closed plastic bag. Seawater samples were subsequently fractionated in order to address the chemical composition of both the particulate and the dissolved phases and the resulting fractions were then analyzed using a targeted metabolomic approach. The chemical pattern showed that specialized metabolites are detected in all environmental compartments (particulate and dissolved phases, Fig. 2A). These detectable concentrations of specialized metabolites in the seawater surrounding the sponges confirm that low amounts of such metabolites (0.03–0.37% of the concentration in the sponge endometabolome, Table 1) are permanently released by *C. crambe* though natural processes, forming the exometabolome. The particulate phase was shown to be slightly enriched in all kinds of metabolites (Σ PGA = 3.54 μM) compared to the dissolved phase (Σ PGA = 2.43 μM). However, a detailed inspection of the chemical patterns obtained for both phases showed that crambescins are roughly equally distributed whereas crambescidins are more associated to the particles (by a factor >2). As a rule, crambescidin-816 constitutes the most abundant compound (32 and 23% of the total abundance in the particulate and dissolved fractions respectively), followed by the crambescins A2–448 and C1–466 (14% and 12% respectively in particles).

### Compounds are released in the seawater through a spherulization process

In order to understand the mechanism of metabolite exudation, we needed to enhance the exudation process through a mechanical stress by gently pressing the sponge without damaging its surface^18^,^37^. As expected, this mechanical stress increased the total amount of specialized metabolites in the particles of the surrounding seawater by a factor 47, compared to a natural condition (Fig. 2A,B). There was a corresponding decrease in the amount of specialized metabolites in the endometabolome of stressed samples, leading to a total amount of PGA of 52 μmol.g sp^−1^ (Table 1).

The particulate matter was subjected to further analyses in order to determine whether these metabolites were associated to bacterial or sponge cells released by the sponge. We quantified total bacteria as well as the already known *C. crambe* associated Betaproteobacterium, in different particulate fractions of seawater samples surrounding both treated and controls. Although the Betaproteobacterium was directly targeted by its specific BET467 probe, we were unable to detect stained cells on the filters (Supplemental Figure S3). Images obtained from probe EUBI-II-III showed a number of stained cells indicating that the lack of signal was not caused by a problem with the CARD-FISH. We observed a small but significant (t-test p < 0.05) decrease in total bacterial numbers of seawater samples surrounding stressed (2.37 ± 0.11 × 10^5^) compared to control (3.31 ± 0.11 × 10^5^) sponges.

**Figure 3:**
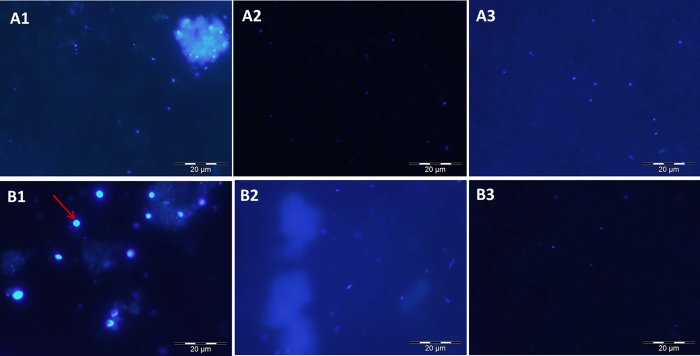
DAPI stained filters observed at 1000x magnification. (**A**) Controls (**B**) stressed treatments -(A1&B1) 3 μm filters; (A2&B2) 0.45 μm filters (A3&B3) 0.2 μm filters. Arrow: nucleus of a sponge cell. Bar: 20 μm.

**Figure 4:**
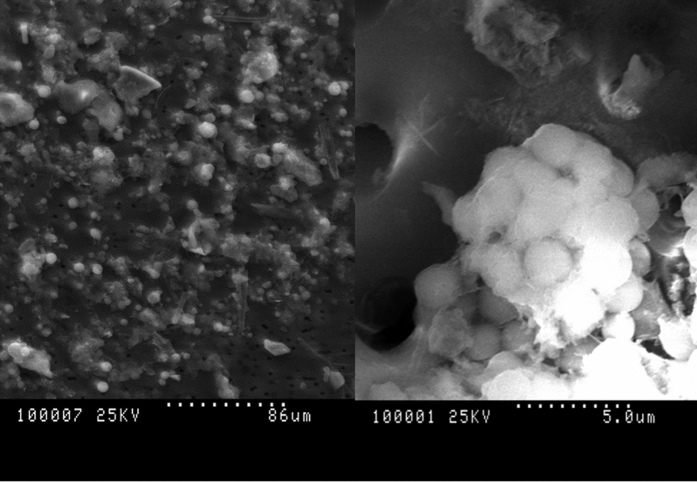
Scanning electron microscope observations of seawater filtered (**A**) Global view upon 3 μm, (**B**) spherulous cells containing spherules (upon 3 μm).

Remarkably, DAPI-stained 3 μm filters (Fig. 3) showed a number of larger cells that were clearly identified as sponge cells by comparing them with previous observations on *C. crambe* tissue samples^34^, and these cells were not observed in all other filters. To further explore the presence and the identity of sponge cells released by *C. crambe*, parallel filters were visualized by Scanning Electron Microscopy. Cells of various shapes and sizes were observed including two types of sponge cells (Fig. 4A). The most common sponge cell type was constituted by around 10 μm-cells filled with small vesicles (1–3 μm; Fig. 4B), previously described as spherulous cells^38^. Many of those spherulous cells were observed lysed, resulting in an important release of small vesicles called spherules (Fig. 4B and supplemental Figure S4) over the entire surface of the filter. Due to their lower size, such spherules can pass through the pores of the largest filter (3 μm) and remain on the 0.45 μm filter as seen on Fig. 4C. This observation is only in accordance with the PGA transported by the spherules.

With all these data in hands we were able to assess the approximate amount of compounds contained in each spherule. Interpreting several images of the filters of diameter 13 mm at 3 μm we were able to count 115 ± 15 spherulous cells in a picture (N_SC/P_) of 0.2 mm × 0.2 mm (S_P_ 0.04 mm^2^). Considering the whole surface of the filter (S_F_ 133 mm^2^) and an inhomogeneous distribution of the cells due to a spiral support for the filter we estimated a value of 1.9 × 10^5^ ± 2.4 × 10^4^ spherulous cells in one filter (N_SC/F_) of 3 μm (Equation 1). Each spherulous cell containing an average of 25 spherules (N_S/SC_)^38^, we could assess the number of spherules in the filter of 3 *μ*m N_S/F_ 4.7 × 10^6^ ± 6.2 × 10^5^.


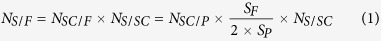


On a 47 mm-filter and with a volume of one liter of filtered seawater, the number of spherules reaches N_S/F_ 5.0 × 10^9^ ± 6.5 × 10^8^ (absence of the spiral effect in this case). For the amount of crambescidin-816 present in the same 3-μm filter, we measured an approximate mass of m_C816/F_ 33 μg using the calibration curve. It means that each spherule contains an approximate mass m_C816/S_ 6.60 × 10^−15^ g of crambescidin 816 thus leading to an approximate value of 8.09 × 10^−18^ mol of crambescidin 816 per spherule or n_C816/S_ 136,000 molecules of crambescidin-816 contained in one spherule of an approximate volume of 4.2 mm^3^ (Equation 2).


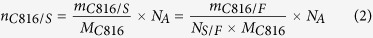


where M_C816_ is the molar mass of crambescidin 816 (M_C816_ 816 g mol^−1^) and N_A_ the Avogadro constant (N_A_ 6.02 × 10^23^ mol^−1^).

### Crambescidin-816 is more toxic than crambescin A2-462 towards tunicate larval development

**Figure 5:**
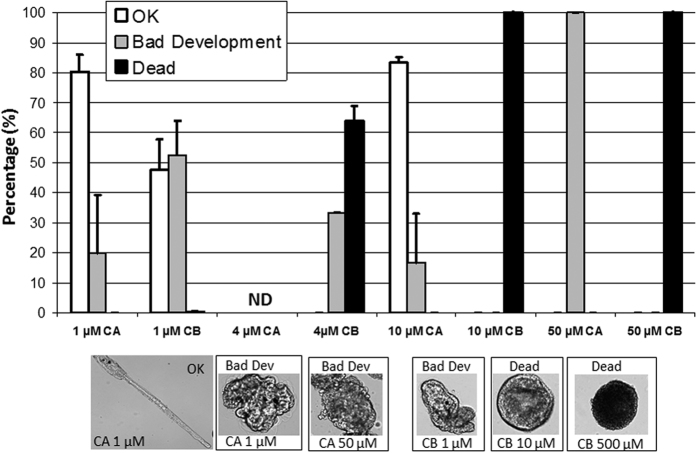
Toxicity of Crambescin A2-462 (CA) and Crambescidin 816 (CB) assessed on ascidian embryos (*Phallusia mamillata*). 3 cultures from 3 different animals for each point (except 4 μM CA was not determined (ND)) with around 100 embryos counted per culture. Paired control cultures (in the presence of 0,1% DMSO) showed 67% of good development (not shown). Grey bars: «Bad development» indicates teratogenicity (i.e. embryonic malformations). White bars: «Dead» is for lysed or uncleaved embryos and indicates cytotoxicity.

We assessed the toxicity of one representative of each family of alkaloids produced by *C. crambe* namely crambescin A2-462 (C462) and crambescidin 816 (C816) on the embryonic development of the solitary tunicate *Phallusia mamillata*. Even if both compounds were found to be toxic (Fig. 5), crambescidin 816 was found to be teratogenic above 1 μM and cytotoxic above 4 μM whereas crambescin A2-462 had no toxicity at 10 μM and was only found to be teratogenic at 50 μM. In order to assess the ecological relevance of these results, we estimated the absolute concentration of C462 and C816 in dissolved and particulate samples from the calibration curve performed for this compound. These two compounds are naturally present in the dissolved/particulate phases at concentrations of 0.20/0.27 μM (C462) and 0.82/1.51 μM (C816), suggesting that teratogenic concentrations of C816 are continuously reached under a particulate form and almost reached under a dissolved form and could prevent any development of the embryo and larvae on the sponge surface. It is noteworthy that our mechanical stress yielded to higher concentrations of C462 in particles (1.84 μM) as well as of C816 in both the dissolved and the particulate compartments (5.13 and 46.40 μM respectively), that would be cytotoxic to tunicate embryos.

## Discussion

Allelochemical interactions between sponges and other organisms remain poorly understood, especially with regards to the mode of action: do sponge allelochemicals act only through contact or are they released in the seawater just like volatile mediators in the air? Our study provides insights into this issue taking the encrusting Mediterranean sponge *Crambe crambe* as a model. To this end we inspected the content and mode of transport of the sponge exometabolome focusing on PGA in the different particulate and dissolved phases. The ecological relevance of the observed mode of transport has been assessed by an ecotoxicological assay on ascidian larval development.

The detection of PGA in the sponge surrounding indicates a continuous transfer of the specialized metabolites from the sponge to the seawater. Larger amounts of PGA being detected in the particulate phase (2.43 > 3.54 μmol.g sp^−1^) we assumed that compounds detected in the dissolved phase derive from the solubilization of the particulate form. Considering a predicted logP, the solubilization of the crambescins (1.96 > logP > 4.29) will likely occur more quickly than for crambescidins (logP > 9.70, Chemdraw software). However, results from a previous experiment showed that this solubilization is still straightforward as the abundance of crambescidin 816 in the particulate fraction decreased by 25% after only one hour and more than 60% after 8 hours (Supplemental, Figure S5). This natural release of specialized metabolites was amplified by our stress, showing a clear link between the endometabolome and the exometabolome as the former decreased by a factor 1.3 (from 71 to 52 μmol.g sp^−1^) while the latter increased by a factor 16.4 (from 5.96 to 97.81 μM). The PGA are clearly exuded by the sponge through a determined ecological mechanism.

The higher concentrations of PGA found in the particles comparing to the dissolved fraction in stressed conditions suggested that specialized metabolites were most probably exuded from the sponge under a particulate form before being dissolved. This observation gave a first insight on the mechanism of exudation and the fate of these specialized metabolites in the marine environment. The results obtained in this study supported that specialized metabolites are stored in sponge cells rather than associated bacteria. Sponge metabolites have been localized in different compartment of the organism, sometimes concentrated on the surface sometimes in the inner compartments. These localizations can explain the absence of fouling on most sponges but also the non-invasion of pathogenic microorganisms. Here we were able to demonstrate an additional and natural phenomenon consisting in the constant release of sponge metabolites in the environment. Looking into the different phases of the surrounding seawater, microscopic inspection of the filters evidenced that the presence of the PGA is strongly correlated to the presence of spherulous cells released by the sponge. This observation is in accordance with the massive release of sponge cells through the exhalent canals recently reported for the encrusting tropical sponge *Halisarca caerulea*^39^, although the authors explained this shedding of cells as a mechanism regulating cellular homeostasis. Previous physiological observations support the hypothesis of spherulous cells release by *C. crambe*. Indeed, in some sponge species, these type of cells have been observed concentrated just beneath the endopinacoderm of the exhalent canals^40^ then passing through the pinacocyte layers of canals^41^ and finally released in the surrounding seawater through exhalent canals^42^.

Specifically, the chemical pattern of the particulate fraction obtained in the present study (concentrations of PGA two to six times higher in the 0.45 μm compared to the 3 μm, Fig. 3) strongly suggests that spherules grouped in the spherulous cells play the role of storage of the specialized metabolites following the results of Uriz and co-workers^38^ which showed antimicrobial activity of spherulous cells in *C. crambe*, albeit with no chemical characterization. Thompson and co-authors^40^ proposed a similar hypothesis on a tubular sponge *Aplysina fistularis* but that study did not show direct proof of the phenomenon. Evidences of the bursting spherulous cells are not only provided by our study but also by Vacelet^42^ and Uriz and co-authors^38^. Images obtained by sponge cross-sections in the latter studies confirmed that spherulous cells bursting did not result from artifacts linked to the filtration of seawater samples under high-vacuum conditions but stems from a natural biochemical process that occurs within the exhalent canals or outside the sponge. Together with our observations these studies lead us to propose that some spherulous cells are involved in the storage and release of spherules in a specific zone of the sponge (the exhalent canal or the ectoderm) while the lipidic spherules themselves concentrate amphiphilic specialized metabolites. By packing several spherules (up to 25 according to Uriz *et al*.^38^) a single spherulous cell could release chemical signals of high intensity. Direct evidences of the presence of metabolites in sponge spherules could be given by maldi-ToF imaging for example.

**Figure 6:**
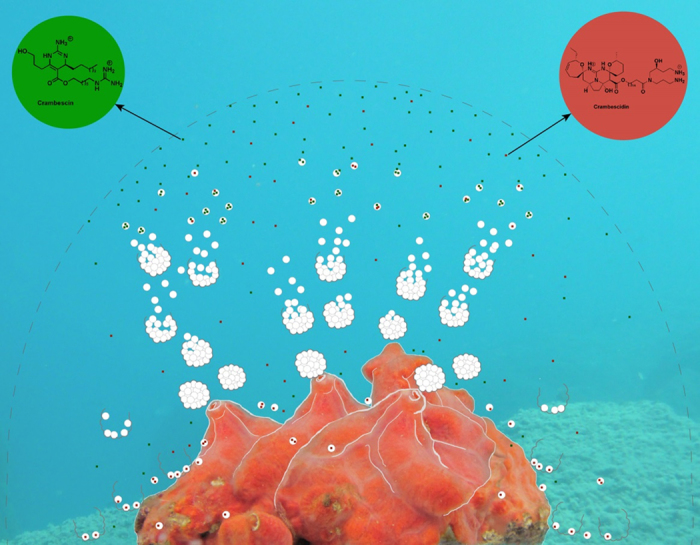
A schematic view of the chemical schield surrounding the encrusting sponge *C. crambe*. ^®^Lisa Ternon.

Diffused in the seawater, allelochemicals would be able to reach chemoreceptors of surrounding organisms either conveyed by the spherules or under a dissolved form. Up to now, there is no information regarding either the mechanism or the kinetic of degradation of these compounds in the marine ecosystem: are they subjected to dispersion, chemical or biochemical transformations? Despite rather fast transformation processes, natural and continuous exudation of spherules would maintain a steady-state concentration of allelochemicals around the sponge, creating a mediation signal around the sponge. A 1 L volume of seawater was collected as close as possible to the encrusting sponge avoiding any contact, leading to an estimation of a chemical shield that would at least correspond to a half-diameter of 20 cm for a sponge of 5 cm square and ~1 g of total dry weight but that could clearly be under-estimated. This chemical shield is due both to the dissolved and the particulate form with suspended spherules (Fig. 6). Due to a natural effect of gravity, spherulous cells and spherules will be deposited onto the sponge surface and its surroundings after expelling by the exhalent canal. The exhalent force being unknown we were not able to measure the actual dispersion of those spherules around the sponge. However, the abundance of spherules and the subsequent chemical effect will most likely follow a decreasing gradient from the sponge to its surrounding substrate. As a consequence, other sessile or slow-moving benthic organisms able to perceive this chemical gradient^16^ might modify their settlement and route.

Interestingly, our study demonstrates that all crambescins and crambescidins participate in the chemical shield. The physico-chemical and subsequent biological features of crambescins and crambescidins are linked to their chemical structure. In particular, crambescidins contain a large lipophilic part (log P = 9.79–10.19) that may anchor these compounds in the spherules. Crambescidins would then be less prone to solubilization and would preferentially remain within the spherules while crambescins (1.96 > logP > 4.29) would more quickly diffuse under a dissolved form. This diverging behavior between the two families of compounds was even also observed by our ecotoxicological assay. Indeed, crambescidin 816 is by far the most toxic compound, a result in accordance to previous studies on human cell lines^26^,^30^,^31^,^43^ whereas crambescin A2 never reached such toxicity. We recently proposed metabolic pathways showing a strong connection between both families of compounds^44^. According to this hypothesis, crambescins and crambescidins share a biosynthetic intermediate, which submitted to different subsequent enzymatic transformations, would lead to increase the concentration of one family of compounds. The activation of one metabolic pathway rather than the other could be tuned accordingly to the presence of a stress. This assumption is highly supported by the chemical pattern of the exometabolome under stressed conditions: in the case of our mechanical stress the chemical halo is enriched in crambescidin 816 by a factor 47 regarding a natural situation and more generally, the crambescicin family represented 47% of the total abundance of allelochemicals in spherules in that context. In case of stress, cytotoxic crambescidins are quickly delivered to the sponge surrounding. By packing up to 130,000 molecules of crambescins 816 (see Results section for estimate), the spherules could deliver a chemical signal of an intensity far exceeding the concentrations we tested on tunicate embryos. Therefore the toxic effects of PGA within spherules being ingested, filtered or simply coming into contact with organs, chemoreceptors or tissues could have been underestimated in this study.

Chemical mediation from *C. crambe* may be supported by a toxicity of contact through spherules settling onto the surface of the sponge and the surrounding substrate, but also by the diffusion of allelochemicals suspended in the water column. The dissemination of dissolved PGA is expected to act as distance chemosensory cues reaching chemoreceptors of various organisms^16^. We believe that this diffused chemical signaling is less toxic than the matching contact signaling, this hypothesis being supported by the low concentration (Σ = 2 μM) of crambescins or crambescidins naturally encountered in the dissolved phase.

With regards to the biosynthetic origin of the PGA, it is now clear, based on the size fractionation and the absence of the bacterial symbionts in the sponge surroundings that these compounds are stored and likely partially produced by sponge cells. Since the turnover of the molecules in the sponge is unknown, and the proposed biosynthetic routes (27) involve polyunsaturated fatty acids, likely to have a microbial origin, involvement of the symbionts in the biosynthesis remains possible.

The diverse chemical strategies developed by *C. crambe*, in particular through the release of cytotoxic compounds, is likely to contribute to this species ecological success in the Mediterranean Sea. *C. crambe* is a very common encrusting sponge and it has recently been proposed that its distribution has extended into the Macaronesian archipelago after frequent exchanges between these maritime areas^45^. Several other sponge species, most of them encrusting, have recently been described as invasive of various marine ecosystems^46^,^47^ increasing substrate coverage by sponge worldwide and in particular in coral reefs^48^,^49^,^50^. Among the factors that could explain this expansion, climate change is likely to induce long-term shift in coral reef ecosystems towards a sponge and/or macroalgae-dominated ecosystems^51^,^52^. Here we propose that the highly bioactive exo-metabolome of some encrusting sponges could also be involved in this ecological success.

## Material and Methods

### Sampling

All underwater experiments were performed by SCUBA diving at 19 m depth in the Rade de Villefranche-sur-Mer, France (Grotte du Lido, Northwestern Mediterranean Sea). Twelve sponge specimens of the encrusting *C. crambe* with areas of approximately 50 cm^2^ (3–5 oscules) were selected for the *in situ* experiments. Natural seawater samples were collected using 1 L plastic bags closed by a zipper lock system at 1 cm above six sponge specimens taking care of not touching the surface of the sponge. After a stress consisting of five moderate fingertip pressures distributed over the sponge surface applied to six other specimens^53^, six stressed seawater samples were also collected. Biomass of the 12 *C. crambe* specimens was harvested with their rocky substrate using a hammer and a chisel and placed in plastic bags underwater. Sponge samples were immediately drained and frozen at −20 °C after collection until chemical treatment while seawater samples were quickly subjected to sequential filtrations for analyses (detailed in metabolomics analyses). One non-stressed and one stressed seawater samples were further divided in several aliquots in order to investigate (i) the presence of the major symbiotic bacterium of *C. crambe*^34^ in the water, (ii) the presence of sponge cells, and (iii) the presence of specialized metabolites in both particulate and dissolved fractions.

### Epifluorescence microscopy and flow cytometry analysis

For epifluorescence microscopy (DAPI staining and CARD-FISH) triplicate water samples (3 from before and 3 after stress) were successively filtered onto 25 mm polycarbonate filters of 3 μm, 0.8 μm and 0.2 μm porosity using 30 mL, 10 mL and 2 mL volumes respectively. Triplicate 2 mL water samples (3 from before and 3 after stress) were fixed with 2% formaldehyde and stored at −20 °C prior to flow cytometry analysis. Finally, a volume of 10 mL of seawater was stored at −20 °C for SEM observations.

Immediately after filtration, polycarbonate filters were placed onto Whatman paper pads saturated with a 4% paraformaldehyde/PBS solution and washed following the basic protocol described in Lam and Cowen^54^, and stored in petri dishes at −20 °C until analysis. Total DAPI-stained cells, cells belonging to the domain Bacteria as well as the specific Betaproteobacteria symbiont cells were specifically stained on all filters according to Croué *et al*.^34^. Briefly, all filters were submitted to double hybridization by CARD-FISH using both the EUBI-II-III probe which targets Bacteria as well as the BET467 probe targeting the Betaproteobacteria symbionts. All filters were counterstained with DAPI. For flow cytometry, samples were thawed at room temperature and stained for 15 min with a SYBR green I (1:400 dilution; Molecular Probes, USA). Cell counts were performed using a FACSCalibur flow cytometer (Becton Dickinson, USA) equipped with an air-cooled argon laser (488 nm, 15 nM) as previously described^55^.

### Scanning Electron Microscopy (SEM) analysis

Samples were fixed with 10 mL of a mixture of glutaraldehyde 25% (2 mL) and cacodylate buffer 0.4 M (8 mL) in a procedure adapted from ref. 56. Samples were then filtered onto 3 *μ*m (diameter 13 mm) polycarbonates membranes and rinsed three times with 0.2 *μ*m filtered seawater. A post-fixation of all filters was performed with 2% OsO_4_ in 0.2 *μ*m filtered seawater. Filters were submitted to successive rinsing: once with filtered seawater, twice with deionized water, three times with ethanol 30%, once with ethanol 50%, three times with successively ethanol 70%, 95% and 100%. The filters were then kept in ethanol 100% during one hour, critical-point-dried, sputter-coated with gold-palladium, and observed under a Hitachi S 570 scanning electron microscope.

### Targeted metabolomic analyses

#### Sample Processing

For targeted metabolomics analysis, a volume of approximately 900 mL of seawater samples was filtered successively through nylon filters of 3.0 *μ*m and 0.45 *μ*m pore-size in order to collect different sizes of the particulate phases. Filters were dried at room temperature and stored refrigerated (4 °C) until further treatment. Filtrates were frozen at −20 °C for further extraction of chemical cues in the dissolved phase.

##### Particulate phase

For the particulate phase, each nylon filter was cut into 23.5 mm pieces and placed into a 20 mL glass vial with 15 mL of a mixture of MeOH/CH_2_Cl_2_ (1:1, v/v) and was sonicated (35 kHz) during 15 min followed by centrifugation at 1600 rpm for 10 min. The filter was then removed from the vial and discarded while the solvent was evaporated until dryness using a SPD111 SpeedVac (Thermosavant, Model RH12-28). The vial content was solubilized in 250 *μ*L of MeOH and filtered through 0.2 *μ*m (PTFE) for UHPLC-HRMS analyses.

##### Dissolved phase

For the dissolved phase, filtrates were defrosted in the dark at room temperature and filtered through octadecyl-bonded silica extraction discs (C_18_ SPE discs, Restek^®^) under vacuum to adsorb dissolved metabolites. The extraction and elution using the C_18_ disc was performed following the procedure provided by the supplier with MeOH as the eluent. The total organic extract was evaporated to dryness in a rotary evaporator and solubilized in 1 mL of MeOH. All extracts were transferred to 7 mL vials and evaporated to dryness using the SpeedVac. Extracts were solubilized in 250 *μ*L of MeOH and transferred to vials prior to UHPLC-HRMS analyses.

##### Biomass

For the sponge samples, all specimens were freeze-dried and ground to obtain a dry powder which was extracted twice with 20 mL of a mixture of MeOH/CH_2_Cl_2_ (1:1, v/v) in an ultrasonic bath (35 kHz) at room temperature. *C. crambe* being an encrusting sponge, its rocky substrate was calcined at 450 °C for 24 h to give access to the sponge organic weight. Crude extracts were dried using a rotary evaporator and then fractionated by solid phase extraction over a 2 g of C_18_ silica packed-column with a step gradient of H_2_O (20 ml), MeOH (20 ml), CH_2_Cl_2_ (20 ml). The methanol fraction was dried using SpeedVac, dissolved at 10 mg/ml and stored at −20 °C until further dilution prior to LC-MS analysis.

#### UHPLC-HRMS data analyses

Prior to the UHPLC-HRMS analysis, all samples were diluted in MeOH (ranging from 1/40 for the sponge samples to ½ for the water and particulate samples) to avoid saturation of the signals. In addition, eight QC samples were prepared by combining 10 μL of each sample in a 2 ml vial and these QC samples were injected every seven samples to allow chromatogram alignment during data treatment. On-line UHPLC-UV-HRMS analysis was performed using a Dionex system Ultimate 3000 equipped with an autosampler and a Dionex Ultimate 3000 diode array dual absorbance wavelength detector (210 and 280 nm), linked to QT of mass spectrometer fitted with an electrospray ionization interface (Bruker Impact II). Mass spectra were recorded in the positive mode. UHPLC separation was achieved on an analytical Nucleodur PolarTec column (100 × 2 mm, 1.8 μm, Macherey Nagel) using a linear elution gradient of H_2_O/MeCN/formic acid to which was added 10 mM of ammonium formate from 80:20:0.1 (v/v/v, isocratic from 0 to 2 min) to 40:60:0.1 (v/v/v, isocratic from 8 to 10 min, flow rate 0.45 mL/min for a total of 14 min. The injected volume was set at 10 μL and detection set at 280 nm. The mass spectrometer analyzer parameters were set as follows: nebulizer sheath gas, N_2_ (2.1 bar); dry gas, N_2_ (8 L/min); capillary temperature, 200 °C; capillary voltage, 2500 V; end plate offset, 500 V; collision gas, He; collision energy, 4 eV. Data were acquired in the 50 to 1200 *m/z* range.

##### UHPLC-HRMS data processing

Following UHPLC-HRMS data acquisition, base peak chromatograms (BPC) were exported as line spectra and converted into the netCDF file format to process the data in centroid mode with XCMS^57^ on R software. The XCMS approach involved peak picking and integration, peak grouping for identification between samples, chromatogram alignment to avoid retention time deviation and at last peak filling to avoid missing values in chromatograms. XCMS generated a matrix (ions/Retention time × sample) that was exported on using Microsoft Excel. The coefficient of variance of the QC samples was 33.

##### Metabolite annotation

Chemical identification was performed only for compounds of interest (crambescins and crambescidins) that have been extensively described in the past. The compounds were then thoroughly identified by comparing their retention time and MS pattern to a standard as well as to the exact molecular weight (Table 2) as previously described^23^,^44^. Because several ion adducts can be detected for a single compound with electrospray ionization, the following mono- and di-charged adducts were also taken into account in the metabolites identification: [M+H]^+^, [M+Na]^+^, [M+NH_4_]^+^, [M+2H]^2+^, [M+2NH_4_]^2+^ which are the most commonly observed in positive electrospray ionization mode with ammonium formate and formic acid as eluents. The theoretical *m/z* values for typical adduct species were compared with the experimental values to ensure the identification of the compounds (once per treatment and fraction and once for the QC samples). For spectra presenting multiple adducts, we summed the area of all adducts in order to obtain the total intensity corresponding to one compound. Analytical blanks confirmed that no memory effects or samples contamination biased our results. The abundance of the specialized metabolites was calculated using a calibration with standards of crambescidin-816 and crambescin-A2-462 (Supplemental Figure S1), assuming all compounds of the same family had similar ionization.

#### Ecotoxicological assays

The tunicate *Phallusia mammillata* was used to assess the toxicity of crambescin A2-462 (C462) and crambescidin 816 (C816) (taken from our compound library). Eggs and sperm of *Phallusia mamillata* were obtained as previously described^58^,^59^. Eggs were then fertilized, washed from sperm and transferred in filtered seawater (SW) + TAPS (pH 8) for culture. 30 min after fertilization, between 50 and 100 zygotes were transferred in SW containing either DMSO only (solvent control) or in SW containing CA and C816 (dissolved from a stock solution in DMSO, max DMSO concentration is 0.1%) for culture during 12 h. Twelve hours after fertilization *Phallusia* embryos are at swimming tadpole stage after hatching. Toxicity was determined by counting the different phenotypes obtained after 12 hours of culture. Three different phenotypes were scored: 1) good development (tadpole with good tail extension); 2) bad development indicating teratogenicity (embryos proceeding through development but displaying either crooked tail (good development until tail extension) or no tail (developmental toxicity before tail extension), or showing no signs of gastrulation or no apparent tissue differentiation); 3) dead indicating cytotoxicity (cell lysis or no cell division). Control cultures in the presence of 0.1% DMSO (solvent control) and 1% DMSO (a concentration of DMSO known to be embryotoxic) were performed for each experiment to control the quality of the embryos used for toxicological testing. Toxicity of the tested compound is established when the test culture shows significantly less embryos with good development (p < 0.05) and significantly more embryos with affected development (in each class: i.e. bad development, dead, p < 0.05). With this protocol, 1% DMSO was found to be toxic to development (good development: 0%; bad development significantly increased) whereas 0.1% was not toxic (Supplemental, Figure S2). All culture conditions were repeated at least three times with embryos coming from at least three different animals.

## Additional Information

**How to cite this article**: Ternon, E. *et al*. Spherulization as a process for the exudation of chemical cues by the encrusting sponge *C. crambe*. *Sci. Rep.* **6**, 29474; doi: 10.1038/srep29474 (2016).

## Supplementary Material

Supplementary Information

## Figures and Tables

**Figure 1:**
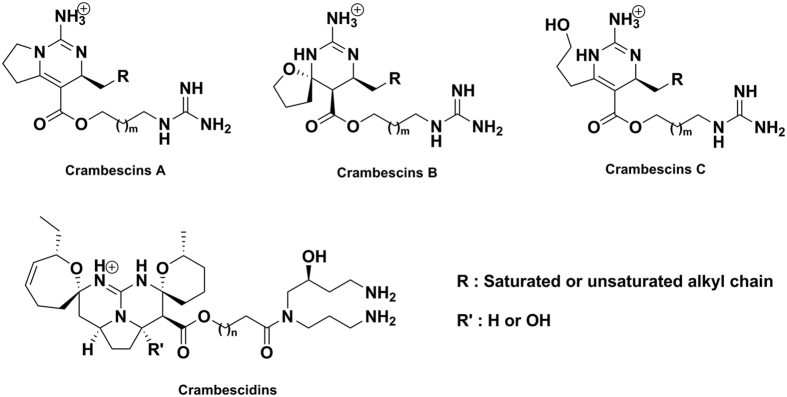
Chemical structures of crambescins and crambescidins with m = 14 for crambescidins, m ≥ 4 and R = C_6_H_13_ for crambescins A1, B1, C1 and m = 2 and R = C_8_H_17_ for crambescins A2, B2 and C2.

**Table 1:** Amount of PGA in the endo-metabolome of *C. crambe* under natural and stressed conditions (μmol.g sp^−1^).

PGA	448	460	462	466	476	480	494	524	538	542	554	800	816	830
Natural	27.4	0.24	1.18	2.94	2.91	1.05	0.20	0.43	1.52	1.51	0.14	7.19	22.3	2.48
Stressed	16.5	0.21	1.07	2.48	2.87	0.84	0.12	0.43	1.49	1.08	0.28	6.35	15.1	1.41

**Table 2:** Theoretical *m/z* and associated molecular formula for quantified PGA from *C. crambe* (derived from ref. 44).

Name of PGA		*m/z* [M+H]^+^	Molecular Formula
Crambescin	A2-448	449.3599	C_24_H_45_N_6_O_2_
	Didehydro A1	461.3599	C_25_H_45_N_6_O_2_
	A1/A2-462	463.3755	C_25_H_47_N_6_O_2_
	A2-476	477.3912	C_26_H_49_N_6_O_2_
	B1/C1-466	467.3704	C_24_H_47_N_6_O_3_
	B1/C1-480	481.3861	C_25_H_49_N_6_O_3_
	B1/C1-494	495.4017	C_26_H_51_N_6_O_3_
	A3-524	525.3912	C_30_H_49_N_6_O_2_
	A3-524	539.4068	C_31_H_51_N_6_O_2_
	B3/C3-542	543.4017	C_30_H_51_N_6_O_3_
	Didehydro C3	555.4017	C_31_H_51_N_6_O_3_
	B3/C3-556	557.4174	C_31_H_53_N_6_O_3_
Crambescidin	800	801.6212	C_45_H_81_N_6_O_6_
	816	817.6161	C_45_H_81_N_6_O_7_
	830	831.6318	C_46_H_83_N_6_O_7_
