# Supplementary material for: Spherulization as a process for the exudation of chemical cues by the encrusting sponge C. crambe
Source: Sci Rep. 2016 Jul 6;6:29474. doi: 10.1038/srep29474 (PMC4933965; doi:10.1038/srep29474)
Supplement: Supplementary Information [file srep29474-s1.pdf]

Spherulization as a process for the exudation of chemical cues by the encrusting sponge *C. crambe*.

Eva Ternon[a*] , Lina Zarate[a,b], Sandrine Chenesseau[c], Julie Croué[d], Rémi Dumollard[b], Marcelino T. Suzuki[d] and Olivier P. Thomas[e,c,f,*]

Supplemental Information

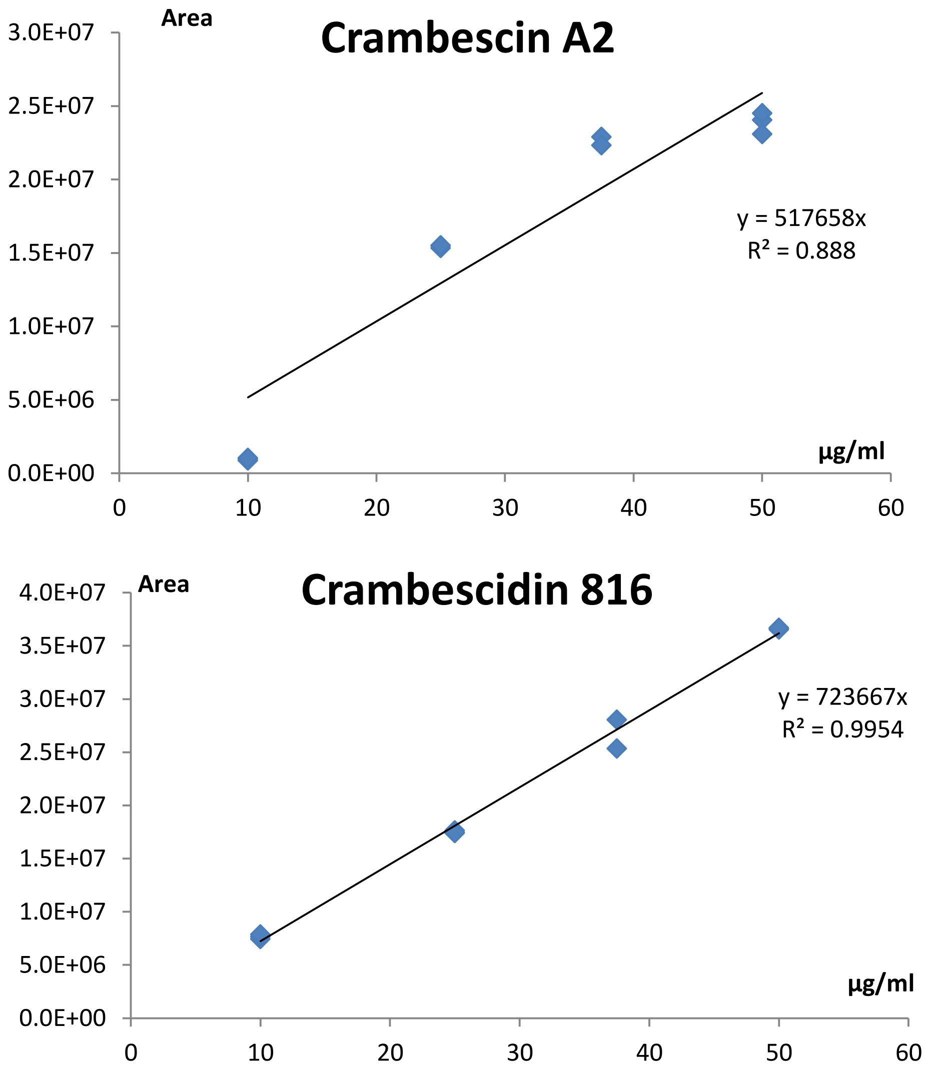

Figure 1. Calibration curve obtained by UHPLC-HRMS for the compounds crambescin A2 and crambescidin 816. Each point corresponds to a triplicate analysis.

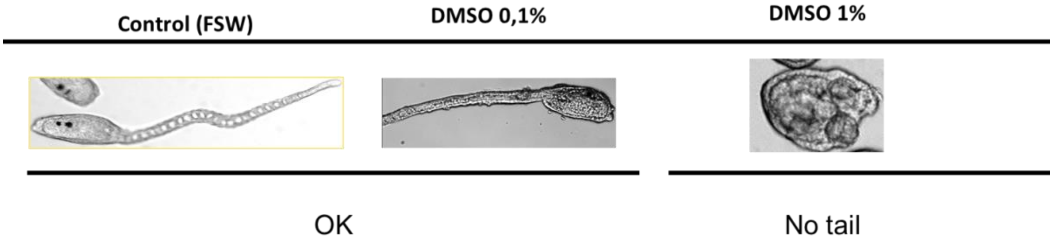

Figure 2. Phenotype obtained for different concentrations of DMSO

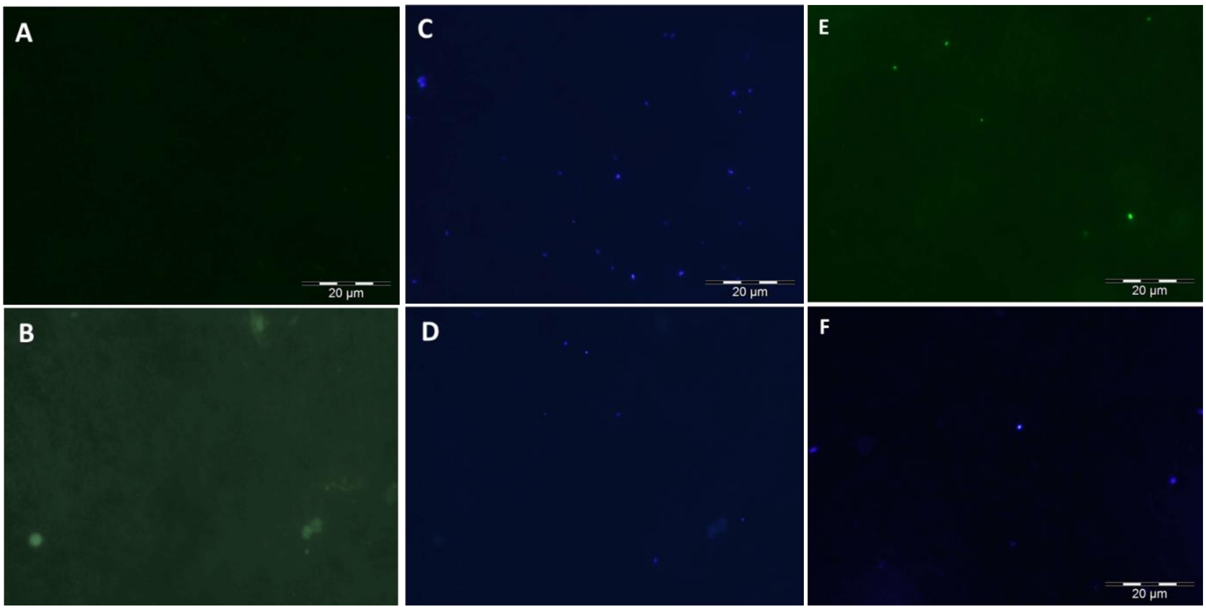

Figure 3. No evidence of Betaproteobacteria was found in the sponge surrounding (0.2 µm filters), targeted with the BET467 probe (A and C) and its corresponding DAPI (C and D). E and F highlight the presence of representatives of marine eubacteria (EU I-II-III probe). Images of the sponge surrounding were taken before (A, C and E) and after (B, D and F) treatment.

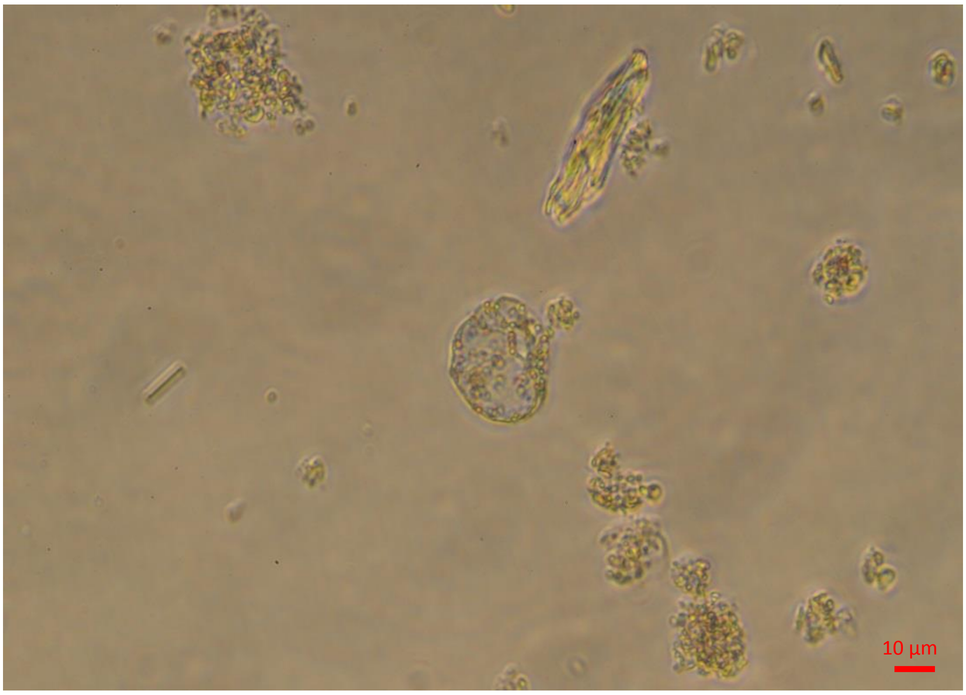

Figure 4. Broken and unbroken spherulous cells and their spherules in stressed seawater samples, under light microscopy (20 x 0.35 pH1).

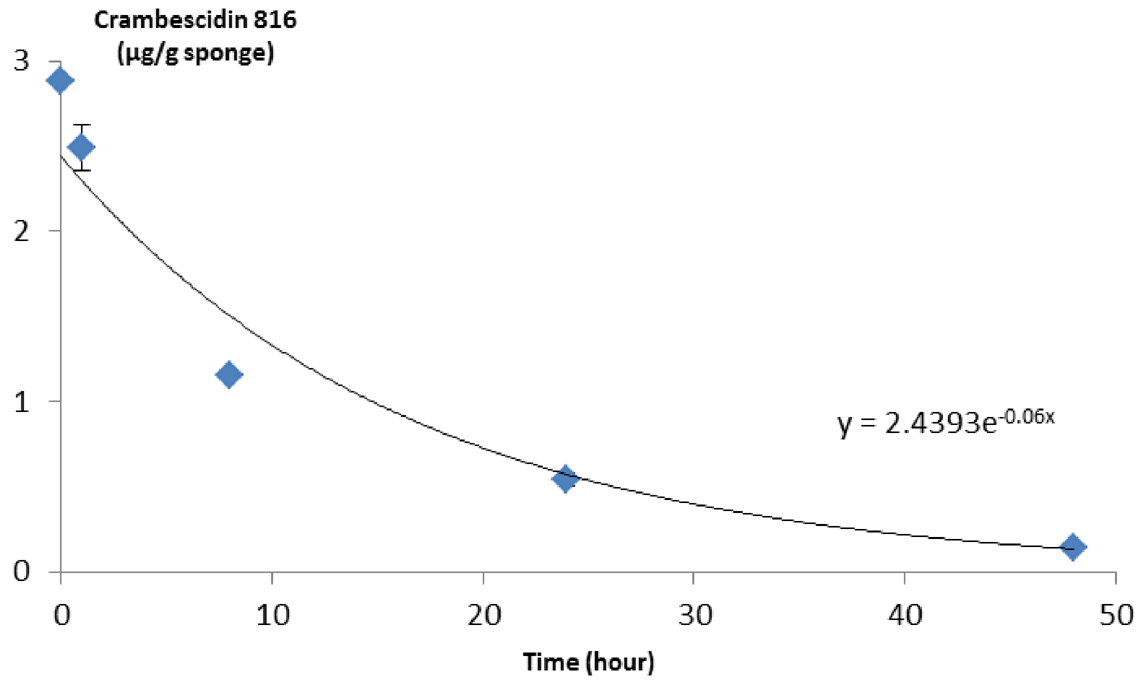

Figure 5. Concentration of crambescidin 816 against time in the particulate phase (0.45 µm) from seawater regularly homogenized and kept in the dark. Error bars represent the variation between triplicates.
